# Locally Recurrent Rectal Cancer: Toward a Second Chance at Cure? A Population-Based, Retrospective Cohort Study

**DOI:** 10.1245/s10434-023-13141-y

**Published:** 2023-02-15

**Authors:** Hidde Swartjes, Jan M. van Rees, Felice N. van Erning, Marcel Verheij, Cornelis Verhoef, Johannes H. W. de Wilt, Pauline A. J. Vissers, Tijmen Koëter

**Affiliations:** 1 Department of Surgery, Radboud Institute for Health Sciences, Radboud University Medical Center, Nijmegen, The Netherlands; 2 Department of Surgical Oncology and Gastrointestinal Surgery, Erasmus MC Cancer Institute, Rotterdam, The Netherlands; 3 Department of Research and Development, Netherlands Comprehensive Cancer Organization, Utrecht, The Netherlands; 4 Department of Surgery, Catharina Hospital, Eindhoven, The Netherlands; 5 Department of Radiation Oncology, Radboud Institute for Health Sciences, Radboud University Medical Center, Nijmegen, The Netherlands; 6 Department of Surgery, Elisabeth-TweeSteden Hospital, Tilburg, The Netherlands

## Abstract

**Background:**

In current practice, rates of locally recurrent rectal cancer (LRRC) are low due to the use of the total mesorectal excision (TME) in combination with various neoadjuvant treatment strategies. However, the literature on LRRC mainly consists of single- and multicenter retrospective cohort studies, which are prone to selection bias. The aim of this study is to provide a nationwide, population-based overview of LRRC after TME in the Netherlands.

**Patients and Methods:**

In total, 1431 patients with nonmetastasized primary rectal cancer diagnosed in the first six months of 2015 and treated with TME were included from the nationwide, population-based Netherlands Cancer Registry. Data on disease recurrence were collected for patients diagnosed in these 6 months only. Competing risk cumulative incidence, competing risk regression, and Kaplan–Meier analyses were performed to assess incidence, risk factors, treatment, and overall survival (OS) of LRRC.

**Results:**

Three-year cumulative incidence of LRRC was 6.4%; synchronous distant metastases (LRRC-M1) were present in 44.9% of patients with LRRC. Distal localization, R1–2 margin, (y)pT3-4, and (y)pN1-2 were associated with an increased LRRC rate. No differences in LRRC treatment and OS were found between patients who had been treated with or without prior n(C)RT. Curative-intent treatment was given to 42.9% of patients with LRRC, and 3-year OS thereafter was 70%.

**Conclusions:**

Nationwide LRRC incidence was low. A high proportion of patients with LRRC underwent curative-intent treatment, and OS of this group was high in comparison with previous studies. Additionally, n(C)RT for primary rectal cancer was not associated with differences in treatment and OS of LRRC.

**Supplementary Information:**

The online version contains supplementary material available at 10.1245/s10434-023-13141-y.

Colorectal cancer (CRC) is responsible for one in ten cancer-related deaths, and about 35% of CRC cases are located in the rectum.^1,2^ After curative resection for primary rectal cancer, a significant proportion of patients will develop locally recurrent rectal cancer (LRRC).^3,4^ Due to the introduction of the total mesorectal excision (TME), high-quality MRI, and (chemo)radiation in the treatment of primary rectal cancer, a major decrease in incidence of LRRC has been accomplished.^5–7^ In current practice, 4–11% of patients are diagnosed with LRRC after curative treatment for primary rectal cancer.^8^ Until 2014, neoadjuvant radiotherapy followed by TME was recommended as treatment for all patients with stage I–III rectal cancer in the Netherlands.^9^ Thereafter, neoadjuvant radiotherapy for cT1–3N0 tumors without a threatened mesorectal fascia (MRF) and limited extramural vascular invasion (EMVI) was abandoned.^10,11^ Previous studies suggested that neoadjuvant radiotherapy for primary rectal cancer might complicate treatment of LRRC, as there are limitations to the reirradiation dose.^8,12^ Additionally, it has been shown that prior neoadjuvant radiation might jeopardize the oncological survival of patients who develop locally recurrent disease.^13^ To this date, no population-based study on LRRC has been performed in the Netherlands since the use of the more restrictive guideline on neoadjuvant treatment. Moreover, only two population-based studies with a focus on LRRC have been performed over the past decades (Detering et al.; Westberg et al.).

The Netherlands Cancer Registry (NCR) has collected high-quality follow-up data of patients diagnosed with primary rectal cancer during the first six months of 2015 only. Using these data, the aim of this study is to provide an overview of incidence, risk factors, treatment, and overall survival (OS) of LRRC after TME in patients diagnosed with nonmetastasized primary rectal cancer.

## Patients and Methods

### Study Design

This retrospective cohort study was conducted including all cases of nonmetastasized primary rectal carcinoma diagnosed in the Netherlands between January and June of 2015 and treated with TME with or without neoadjuvant (chemo)radiation (n(C)RT).

Data on diagnosis, characteristics, and treatment of primary rectal cancer, and diagnosis and treatment of LRRC, were acquired through the Netherlands Cancer Registry (NCR). This registry is managed by the Netherlands Comprehensive Cancer Organization (IKNL) and includes all cancer diagnoses in the Netherlands since 1989. Trained data managers collect general and tumor-specific information from electronic patient files in hospitals. Detailed data regarding the diagnosis and the treatment of local, nodal, and distant recurrences were added to the NCR in 2019. Usually, the NCR does not collect clinical follow-up data. However, aimed to assess outcomes such as disease recurrence, these data were retrieved retrospectively for patients diagnosed in the first six months of 2015. Vital status was updated with the Dutch personal records database on 1 February 2021 for this study. Data on ethnicity and race are not registered in the NCR. The conduct of this study was approved by the ethical committee of the NCR.

Primary rectal cancer stage was reported differently for patients who did and did not receive neoadjuvant treatment. Tumor regression due to neoadjuvant treatment is common in patients who received neoadjuvant treatment. To reflect cancer stage at diagnosis as accurately as possible, clinical cancer stage was reported in these patients. In patients with primary rectal cancer who did not receive neoadjuvant treatment, pathological stage was reported. If clinical cancer stage was missing, pathological cancer stage was used, regardless of neoadjuvant treatment status.

Patients were excluded from this study when the primary rectal tumor was not resected (e.g., watch and wait) or when the primary rectal tumor was resected endoscopically (e.g., during colonoscopy, transanal endoscopic microsurgery, or transanal minimally invasive surgery).^4^

According to ICD-O-3 coding, rectal cancer was defined as C20.9 localization and tumor morphology was defined as nonmucinous adenocarcinoma (8140-8389), mucinous adenocarcinoma (8470 and 8480), signet cell carcinoma (8490), and other.^14^ Low anterior resection (LAR), abdominoperineal resection (APR), and total colectomy were regarded as TME.

Distance between tumor and anal verge was based on MRI assessment. An R1 resection was defined as a resection margin of less than 1 mm.

By consensus, locally recurrent rectal cancer (LRRC) is defined as recurrent rectal cancer within the pelvis.^4^ In the NCR, recurrences of rectal cancer were coded according to the seventh edition of the TNM Classification of Malignant Tumors,^15^ but the exact localization was not registered. Therefore, all nodal recurrences were retrospectively reviewed in the electronic patient files. Nodal recurrences between the aortic bifurcation and the femoral arteries were regarded as intrapelvic, and consequentially defined as LRRC. If distant metastases were present at diagnosis of the local recurrence, LRRC was defined as LRRC with synchronous distant metastases (LRRC-M1). If not, the local recurrence was labeled LRRC-M0.

Curative-intent treatment of LRRC was defined as LRRC resection for LRRC-M0 patients, and LRRC resection with metastasectomy/radiofrequency ablation for LRRC-M1 patients. Palliative treatment of LRRC was defined as systemic therapy (i.e., chemotherapy or hormonal therapy) and/or radiotherapy without LRRC resection. Best supportive care was defined as symptom treatment only.

During the inclusion period of this study, no recommendation on the treatment of LRRC was stated in the national guideline, except that it should take place in a medical center with expertise in the treatment of LRRC.^16^ There was a recommendation in place in this guideline on the frequency and manner of CRC follow-up.

This study has been listed in the ClinicalTrials.gov registry (NCT05475301).

### Statistical Analyses

The risk of diagnosis of LRRC during rectal cancer follow-up is a situation of competing risks: when a patient dies before development of LRRC, death is a competing risk to LRRC diagnosis. Likewise, as only the first detected recurrence was registered in the NCR, diagnosis of isolated distant metastases was another competing risk to LRRC diagnosis. In this situation, statistical methods incorporating competing risks in the analysis are superior to the more general Kaplan–Meier and Cox regression analyses.^17^ Thus, cumulative incidence was estimated using competing-risk cumulative incidence analyses, and between-group differences were compared using Gray’s test.^18^ Risk factors were identified using univariable and multivariable competing risk regression analyses using complete cases, according to the cause-specific hazard model. Covariates were grouped into categorical variables, and were included in the multivariable model when *p* < 0.10 on univariable analyses. To deal with missing data and minimize selection bias, covariates with > 5% missing values were not included in the main multivariable model. An exploratory multivariable model including covariates with > 5% missing data and *p* < 0.10 on univariable analysis was presented alongside the main multivariable model, to be able to weigh the outcomes of the main multivariable model. Nonetheless, these results should be interpreted with caution due to the increased risk of selection bias. Multiple imputation could not be performed, as missing data in the NCR are generally not missing at random, but due to logistic reasons in data registration in specific centers. Median survival and overall survival (OS) were estimated using the Kaplan–Meier method, and between-group differences were compared using the log-rank test. Between-group differences in categorical data were compared using the Fisher’s exact test, and between-group differences in numerical data were compared using the independent *t*-test. *p* < 0.05 was regarded as statistically significant. All statistical analyses were performed using R version 4.1.3 in combination with the “cmprsk” and “survival” package.

## Results

### Patient and Tumor Characteristics

Patients with primary rectal cancer treated without resection (*N* = 218) and by endoscopic resection (*N* = 248) were excluded from the base study population (*N* = 1900). Additionally, patients (*N* = 3) without follow-up data after the primary rectal cancer resection were excluded. In total, 1431 patients with primary nonmetastasized rectal cancer were included in the present study. No patients in the study population had multiple synchronous rectal tumors.

Patient and tumor characteristics are presented in Table 1. The median age of the included primary rectal cancer patients was 67 years [interquartile range (IQR) 61–73 years]. Almost two-thirds (*N* = 939, 65.6%) of the patients with primary rectal cancer were male. Stage III primary rectal cancer represented the largest group of patients (*N* = 776, 54.2%). N(C)RT was given to 12.4%, 56.7%, and 81.6% of patients with stage I, stage II, and stage III rectal cancer, respectively.

**Table 1:** Patient and tumor characteristics of patients with primary rectal cancer

Characteristics	*N* (%) or mean [IQR]
Age	67 [61–73]
*Sex*	
Male	939 (65.6)
Female	492 (34.4)
*Stage*	
I	339 (23.7)
II	314 (21.9)
III	776 (54.2)
Unknown/missing	2 (0.1)
*Pathological T stage*	
(y)pT0	114 ( 8.0)
(y)pT1	156 (10.9)
(y)pT2	495 (34.6)
(y)pT3	617 (43.1)
(y)pT4	40 (2.8)
Unknown/missing	9 (0.6)
*Pathological N stage*	
(y)pN0	994 (69.5)
(y)pN1	305 (21.3)
(y)pN2	127 (8.9)
Unknown/missing	5 (0.3)
*Distance from anal verge*	
0–5 cm	455 (31.8)
5.1–10 cm	593 (41.4)
10.1–15 cm	322 (22.5)
> 15 cm	29 (2.0)
Unknown/missing	32 (2.2)
*Resection type*	
LAR	1023 (71.5)
APR	403 (28.2)
Total colectomy	5 (0.3)
*Morphology*	
Nonmucinous adenocarcinoma	1351 (94.4)
Mucinous adenocarcinoma	68 (4.8)
Signet cell carcinoma	10 (0.7)
Other	2 (0.1)
*Lymphovascular invasion*	
No	951 (66.5)
Yes	244 (17.1)
Unknown/missing	236 (16.5)
*Differentiation grade*	
Good differentiation	38 (2.7)
Moderate differentiation	1130 (79.0)
Poor differentiation	75 (5.2)
Unknown/missing	188 (13.1)
Resection margin	
R0	1369 (95.7)
R1–2	57 (4.0)
Unknown/missing	5 (0.3)
*Circumferential margin*	
Radical (> 1 mm)	1254 (87.6)
Irradical (0–1 mm)	72 (5.0)
Unknown/missing	105 (7.3)

*IQR* interquartile range, *LAR* low anterior resection (including Hartmann procedure), *APR* intersphincteric and extralavator abdominoperineal resection

### Incidence of LRRC

**Fig. 1:**
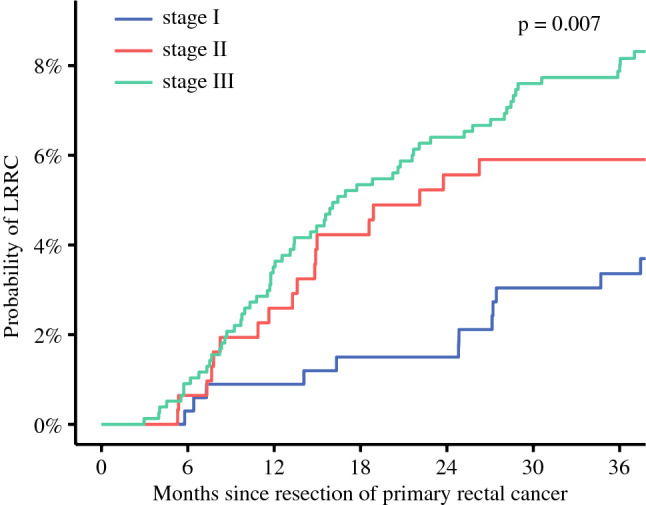
Cumulative incidence plot of LRRC, stratified stage of primary rectal cancer

Median clinical follow-up time after TME was 42.5 months (IQR 37.0–46.1 months). In total, 98 patients were diagnosed with LRRC. Of these local recurrences, 54 (55.1%) were diagnosed without synchronous distant metastases (LRRC-M0) and 44 (44.9%) were diagnosed with synchronous distant metastases (LRRC-M1). Median age at diagnosis of LRRC was 70 years (IQR 63–76 years). One-year and 3-year cumulative incidence of LRRC was 2.7% and 6.4%, respectively. Three-year cumulative incidence of LRRC was 3.4%, 5.9%, and 7.9% for patients with stage I, stage II, and stage III primary rectal cancer, respectively (Fig. 1, *p* = 0.007).

**Fig. 2:**
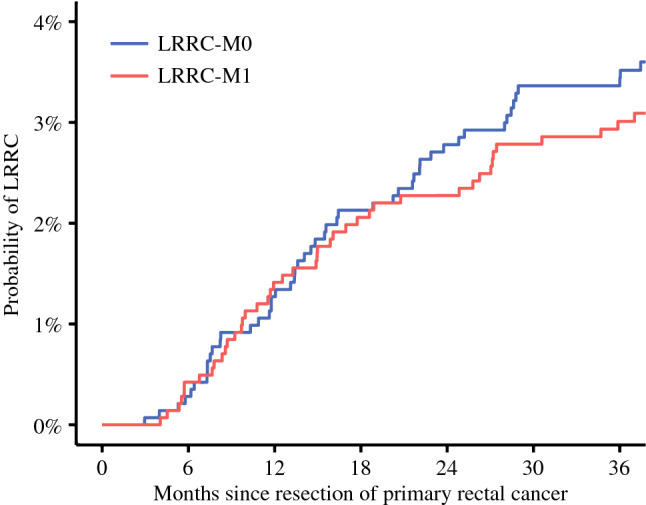
Combined cumulative incidence plot of LRRC-M0 and LRRC-M1

Three-year cumulative incidence of LRRC-M0 was 3.4% and of LRRC-M1 was 3.0% (Fig. 2). Most patients with LRRC-M1 presented with synchronous distant metastases in a single organ (*N* = 28, 63.6%). Presentation with synchronous distant metastases in two sites (*N* = 11, 25.0%), three sites (*N* = 4, 9.1%), and four sites (*N* = 1, 2.3%) was less common.

Of the 98 patients with LRRC, 52 (53.1%) had received n(C)RT for primary rectal cancer; this was equally distributed between patients with LRRC-M0 (55.6%) and patients with LRRC-M1 (50.0%, *p* = 0.685).

### Risk Factors for LRRC

A distance of ≤ 5 cm between the tumor and the anal verge [hazard ratio (HR) 1.6, 95% confidence interval (CI) 1.1–2.4, *p* = 0.028], an incomplete resection margin (HR 4.1, 95% CI 2.3–7.3, *p* < 0.001), pT3–4 tumors (HR 2.4, 95% CI 1.5–3.8, *p* < 0.001) and pN1–2 lymph nodes (HR 2.3, 95% CI 1.5–3.5, *p* < 0.001) were identified as independently associated with LRRC in the main multivariable analysis (Table 2, *N* = 1380 included cases). Additional adjustment for lymphovascular invasion status (HR 1.5, 95% CI 0.92–2.6, *p* = 0.102) and good-to-moderate versus poor tumor differentiation (HR 1.6, 95% CI 0.78–3.4, *p* = 0.275) did not show a significant association between one of these two covariates and LRRC risk (*N* = 1001 included cases, Supplementary Table 1).

**Table 2:** Univariable and multivariable competing risk regression according to the cause-specific hazard method output for the risk of LRRC

	3-Year LRRC estimate, %	Univariable HR (95% CI) *N* = 1431	*p*	Multivariable HR (95% CI), *N* = 1380	*p*
*Age*					
< 70 years	6.0	Reference			
≥ 70 years	7.0	1.2 (0.80–1.8)	0.377		
*Sex*					
Male	6.4	Reference			
Female	6.3	1.1 (0.71–1.6)	0.755		
*Distance to anal verge*					
≥ 5.1 cm	5.6	Reference		Reference	
< 5 cm	8.1	1.6 (1.0–2.3)	0.030	1.6 (1.1–2.4)	0.028
*Resection margin*					
R0	5.6	Reference		Reference	
R1–2	23.0	7.1 (4.1–12)	< 0.001	4.1 (2.3–7.3)	< 0.001
*Resection type*					
LAR	6.3	Reference			
APR	6.6	1.3 (0.82–1.9)	0.303		
*Morphology*					
Nonmucinous adenocarcinoma	6.2	Reference			
Mucinous adenocarcinoma, signet cell carcinoma, and other	8.8	1.7 (0.84–3.6)	0.138		
*Pathological tumor stage*					
(y)pT0–2	3.6	Reference		Reference	
(y)pT3–4	9.6	3.4 (2.2–5.3)	< 0.001	2.4 (1.5–3.8)	< 0.001
*Pathological nodal stage*					
(y)pN0	4.6	Reference		Reference	
(y)pN1–2	10.6	3.1 (2.1–4.7)	< 0.001	2.3 (1.5–3.5)	< 0.001

Covariate selection for the multivariable model was based on *p* < 0.10 in univariable analyses. *p*-Values < 0.05 were regarded as statistically significant

*HR* hazard ratio, *CI* confidence interval, *LAR* low anterior resection (including Hartmann procedure), *APR* intersphincteric and extralavator abdominoperineal resection

### Treatment of LRRC

**Fig. 3:**
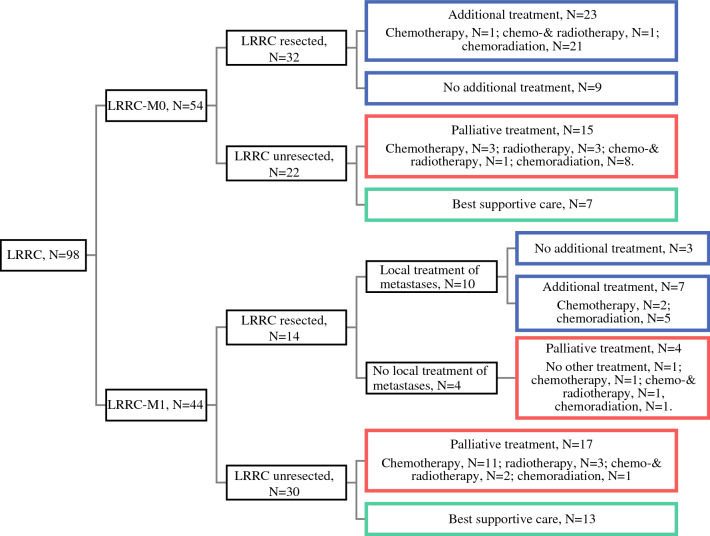
Treatment of patients with LRRC-M0 and LRRC-M1. Blue box, curative-intent treatment. Red box, palliative treatment. Green box, best supportive care

In total, 42 patients with LRRC (42.9%) received curative-intent treatment, 36 patients with LRRC (36.7%) received palliative treatment, and 20 patients with LRRC (20.4%) received best supportive care (Fig. 3).

Median age at LRRC diagnosis was 65 years in the curative-intent treatment group, while it was 71 years and 74 years in the palliative and best supportive care group, respectively (*p* = 0.003). Patients with LRRC-M0 were more often treated with curative-intent treatment than patients with LRRC-M1 (59.3% versus 22.7%, respectively; *p* = 0.001). In 23 of 42 patients with LRRC treated with curative intent (54.8%), a complete resection margin was reached; an incomplete resection margin was present in 11 patients with LRRC (26.2%), and in 8 patients with LRRC (19.0%) data on the resection margin were not reported.

N(C)RT was given to 24 of 98 patients with LRRC (24.5%) during LRRC treatment. The proportion of N(C)RT for LRRC (i.e., reirradiation) was similar in patients who did and those who did not receive prior n(C)RT for primary rectal cancer (23.9% versus 25.0%, respectively; *p* = 1).

### Survival of LRRC

**Fig. 4:**
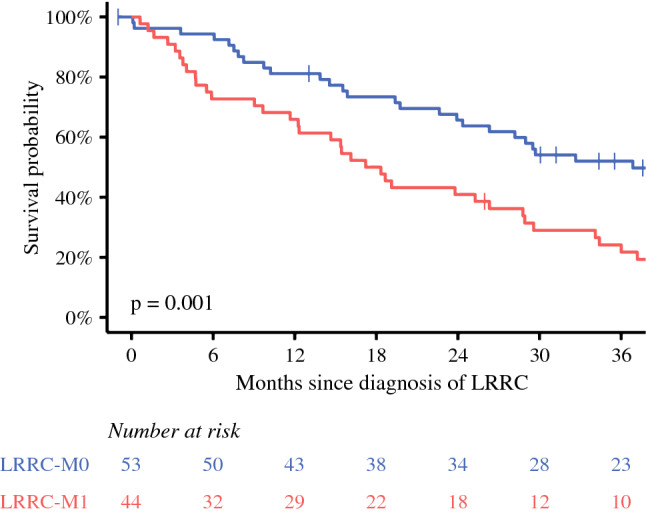
Overall survival plot, stratified for LRRC-M0 and LRRC-M1

Median OS of patients with LRRC was 28.2 months (95% CI 19.4–36.0 months). One- and 3-year OS of patients with LRRC was 74% (95% CI 66–84%) and 39% (95% CI 31–51%), respectively. Median OS of patients with LRRC-M0 was 36.9 months (95% CI 26.3 months, upper not reached). Patients with LRRC-M0 had a 1-year OS of 81% (95% CI 71–92%) and a 3-year OS of 52% (95% CI 40–68%). Median OS of patients with LRRC-M1 was 17.8 months (95% CI 12.3–28.9 months), and 1- and 3-year OS was 66% (95% CI 53–82%) and 24% (95% CI 14–41%), respectively. OS of patients with LRRC-M1 was significantly worse compared with patients with LRRC-M0 (*p* = 0.001, Fig. 4).

There was no difference in OS between the patients diagnosed with LRRC within the first year after resection of primary rectal cancer (*N* = 38, median OS 18.9 months, 95% CI 15.4–48.2 months) and the patients diagnosed with LRRC after the first year since resection of primary rectal cancer (*N* = 60, median OS 29.0 months, 95% CI 23.9–38.6 months, *p* = 0.792).

Median OS of patients with LRRC who received prior n(C)RT for primary rectal cancer was 23.9 months (95% CI 22.6–51.4 months), while median OS of patients with LRRC who did not receive prior n(C)RT was 29.0 months (95% CI 15.4–36.9 months, *p* = 0.177).

**Fig. 5:**
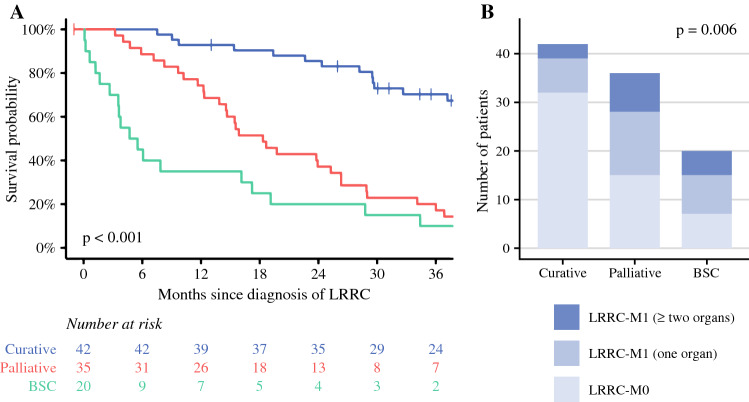
Overall survival plot, stratified for treatment groups (**A**) complemented by a stacked bar chart displaying the distribution of patients with LRRC-M0, patients with LRRC-M1 with synchronous metastases in one organ/site only, and patients with LRRC-M1 with synchronous metastases in two or more organs/sites within and across the treatment groups (**B**)

After curative-intent treatment, patients with LRRC showed a 3-year OS of 70% (95% CI 58–86%, Fig. 5A). The curative-intent treatment group consisted of 76.2% of patients with LRRC-M0 (Fig. 5B). Patients with LRRC treated with palliative treatment had a 3-year OS of 20% (95% CI 10–39%), while patients with LRRC who received best supportive care showed a 3-year OS of 10% (95% CI 2.7–37%, *p* < 0.001).

Three-year OS of the 23 patients with LRRC with complete resection margins after curative-intent treatment was 87% (95% CI 73–100%), while this was 61% (95% CI 38–100%) for the 11 patients with LRRC in whom no complete resection margin was reached.

## Discussion

This population-based, retrospective cohort study conducted with recent nationwide data showed that 3-year cumulative incidence of LRRC in the Netherlands after TME was 6.4% after a median follow-up of 42.5 months. The proportion of patients with LRRC who presented with synchronous metastases (LRRC-M1) was 44.9%, and 55.1% of patients with LRRC presented with LRRC-M0. Tumor localization within ≤ 10 cm of the anal verge, a microscopically incomplete resection margin, (y)pT3–4, and (y)pN1–2 were associated with increased risk for LRRC. Curative treatment was given to 42.9% of patients with LRRC, and 3-year OS after curative-intent treatment of LRRC was 70% (95% CI 58–86%).

The 6.4% 3-year cumulative incidence of LRRC after TME found in the present study is comparable to LRRC rates found in other population-based studies and large randomized controlled trials conducted in the TME era.^19–23^ The 3-year cumulative incidence of LRRC was higher than the 3-year cumulative incidence of locoregionally recurrent colon cancer in the same population.^24^ The proportion of patients diagnosed with LRRC (44.9%) was comparable with other studies, such as a cross-sectional, population-based study from the Netherlands with data from 2011, in which 41% of patients with LRRC presented with distant metastases before or simultaneously with the LRRC diagnosis after curative resection of primary rectal cancer.^25^ Nonetheless, the present study demonstrated a benefit in OS of patients with LRRC-M0 versus patients with LRRC-M1, incorporating patients who were treated with palliative treatment and best supportive care. The sample size of the present study did not allow for a comparison of OS between curatively treated patients with LRRC-M0 and those with LRRC-M1, but a large recent single-center cohort study indicated that curatively treated patients with LRRC-M0 might have a better OS than curatively treated patients with LRRC-M1, because curatively treated patients with LRRC-M1 have worse disease-free and metastasis-free survival.^26^

Previously identified independent risk factors for LRRC were confirmed by the present study (i.e., pT stage, pN stage, resection margin, and distance between tumor and anal verge).^27^ Previous studies have additionally identified both lymphovascular invasion—in particular extramural venous invasion—and poor tumor differentiation as independent risk factors for LRRC development,^28^ but due to a vast amount of missing data in both covariates, they could not be included in the main multivariable model without risking the introduction of selection bias. After including them in an exploratory analysis for both parameters hazards ratios above 1 were observed, but they did not reach statistical significance. This is probably due to the small sample size.

Outcomes of LRRC were superior to those of other population-based studies. Detering et al. reported that 29% of patients with LRRC in their cross-sectional study in the Netherlands received treatment with curative intent, of whom only 55% were treated with a surgical resection in the end.^25^ Moreover, 2-year OS of patients with resected LRRC was only 30%. In the present study, curative-intent treatment was given to 42.9% of patients with LRRC. Spectacularly, 3-year OS survival of these patients was 70%. The enormous difference in rates of resections between Detering et al. and the present study is remarkable, but could possibly be attributed to the increased centralization of LRRC treatment in combination with the introduction of induction chemotherapy.^16,29^ Westberg et al. conducted a population-based study including patients with LRRC after resection of primary rectal cancer between 1995 and 2002 in Sweden, which is methodologically more in line with the present study. In their study, 35.0% of patients with LRRC-M0 received curative-intent treatment, while 45.3% received palliative care and 19.7% were treated with best supportive care,^30^ which is comparable to the distribution of treatment types in the present study. However, it should be taken into account that Westberg et al. included only patients with LRRC-M0, who are more likely to receive curative-intent treatment as shown by the present study. No OS statistic of the curative-intent treatment group as defined by the present study was reported by Westberg et al., but it was reported that the 3-year OS of patients who had underwent a R0 resection for LRRC was 56%. The 3-year OS of the 23 patients with LRRC who underwent a R0 resection was as high as 87% in the present study. Abovementioned comparisons emphasize that the advancements in treatment of LRRC (e.g., increased centralization, increased use of intraoperative radiotherapy, and induction therapy) over the past two decades probably had a major beneficial influence on outcomes of LRRC.^31,32^

No difference in reirradiation rate was found in the present study between LRRC patients who had received prior n(C)RT for primary rectal cancer and those who had not. The Dutch TME trial showed that the OS of patients with LRRC who had received neoadjuvant radiotherapy and TME for primary rectal cancer was significantly worse than the OS of patients with LRRC who received had TME only (median OS 6.1 versus 15.9 months, *p* = 0.008).^13^ In the present study, there was no difference in median OS between patients with LRRC who received n(C)RT for primary rectal cancer and those who did not (23.9 versus 29.0 months, respectively; *p* = 0.178), showing that the impact of prior n(C)RT on recurrent disease management and subsequent outcomes of LRRC is of limited clinical relevance.^33^

The absolute difference between the OS of LRRC found in the Dutch TME trial and the present study presumably reflects the increased quality of medical imaging and treatment of LRRC. Hopefully, the results of the ongoing PELVEX II trial will increase the proportion of LRRC patients with R0 resections,^34^ which could have a beneficial effect on OS of LRRC in general.

This study has several limitations. First, 5-year cumulative incidence and OS rates could not be reported due to the limited follow-up of the study. However, as 89–93% of local recurrences are diagnosed within the first three years after resection of primary rectal cancer, the relevance of the 3-year cumulative incidence must not be underestimated.^13,35^ Second, due to the combination of a 6-month inclusion period and a relatively low incidence of LRRC, 98 patients with LRRC were included. This is a relatively modest sample size of patients with LRRC in comparison with cohort studies focusing on treatment of LRRC.^26,36,37^ However, these studies are not of a population-based nature and the results from these studies are more prone to selection bias than the results from our study. Third, clinical data collection was ended after diagnosis of the first recurrence, preventing estimation of disease-free survival after LRRC treatment or rerecurrence rates.

## Conclusions

This population-based, retrospective cohort study showed that high rates of curative-intent treatment for LRRC were given and OS after curative-intent treatment was very respectable. Additionally, patients with LRRC treated with or without neoadjuvant (chemo)radiation for primary rectal cancer showed few differences in the management of their recurrent disease and no differences in OS.

## Supplementary Information

Below is the link to the electronic supplementary material.

Supplementary file1 (DOCX 19 kb)
