# Supplementary material for: Locally Recurrent Rectal Cancer: Toward a Second Chance at Cure? A Population-Based, Retrospective Cohort Study
Source: Ann Surg Oncol. 2023 Feb 15;30(7):3915–24. doi: 10.1245/s10434-023-13141-y (PMC10250482; doi:10.1245/s10434-023-13141-y)
Supplement: Supplementary file 1 — Supplementary file1 (DOCX 19 kb) [file 10434_2023_13141_MOESM1_ESM.docx]

**SUPPLEMENTARY MATERIAL**

**Supplementary table 1 – Univariable and multivariable competing risk regression according to the cause-specific hazard method output for the risk of LRRC.**

|  | 3-year LRRC estimate, % | Univariable  HR (95% CI)  N=1,431 | *P* | Multivariable  HR (95% CI),  N=1,001 | *P* |
| --- | --- | --- | --- | --- | --- |
| Age |  |  |  |  |  |
| <70 years | 6.0 | reference |  |  |  |
| ≥70 years | 7.0 | 1.2 (0.80; 1.8) | 0.377 |  |  |
| Sex |  |  |  |  |  |
| Male | 6.4 | reference |  |  |  |
| Female | 6.3 | 1.1 (0.71; 1.6) | 0.755 |  |  |
| Distance to anal verge |  |  |  |  |  |
| ≥5.1 cm | 5.6 | reference |  | reference |  |
| <5 cm | 8.1 | 1.6 (1.0; 2.3) | 0.030 | 1.6 (1.0; 2.5) | 0.050 |
| Resection margin |  |  |  |  |  |
| R0 | 5.6 | reference |  | reference |  |
| R1–2 | 23.0 | 7.1 (4.1; 12) | <0.001 | 3.0 (1.5; 6.0) | 0.002 |
| Resection type |  |  |  |  |  |
| LAR | 6.3 | reference |  |  |  |
| APR | 6.6 | 1.3 (0.82; 1.9) | 0.303 |  |  |
| Morphology |  |  |  |  |  |
| Non-mucinous adenocarcinoma | 6.2 | reference |  |  |  |
| Mucinous adenocarcinoma, signet cell carcinoma and other | 8.8 | 1.7 (0.84; 3.6) | 0.138 |  |  |
| Pathological tumor stage |  |  |  |  |  |
| (y)pT0–2 | 3.6 | reference |  | reference |  |
| (y)pT3–4 | 9.6 | 3.4 (2.2; 5.3) | <0.001 | 1.7 (1.0; 2.9) | 0.046 |
| Pathological nodal stage |  |  |  |  |  |
| (y)pN0 | 4.6 | reference |  | reference |  |
| (y)pN1–2 | 10.6 | 3.1 (2.1; 4.7) | <0.001 | 1.8 (1.0; 2.9) | 0.033 |
| Lymphovascular invasion |  |  |  |  |  |
| No | 6.0 | reference |  | reference |  |
| Yes | 11.2 | 2.4 (1.6; 3.8) | <0.001 | 1.5 (0.92; 2.6) | 0.102 |
| Differentiation grade |  |  |  |  |  |
| Good-moderate | 5.9 | reference |  | reference |  |
| Poor | 14.9 | 2.7 (1.4; 5.1) | 0.002 | 1.6 (0.78; 3.4) | 0.275 |
|  |  |  |  |  |  |
|  |  |  |  |  |  |

Covariate selection for the multivariable model was based on p<0.10 in univariable analyses. P-values <0.05 were regarded as statistically significant. HR; hazard ratio. CI; confidence interval. LAR; low anterior resection (including Hartmann procedure). APR; intersphincteric and extralavator abdominoperineal resection.
